# Study of Structural and Optical Properties of Titanate Nanotubes with Erbium under Heat Treatment in Different Atmospheres

**DOI:** 10.3390/ma16051842

**Published:** 2023-02-23

**Authors:** Gelson L. C. Rodrigues, Tainara G. de Oliveira, Suziete B. S. Gusmão, Odair P. Ferreira, Thiago L. Vasconcelos, Yuset Guerra, Raquel Milani, Ramón Peña-Garcia, Bartolomeu C. Viana

**Affiliations:** 1 Federal Institute of Piauí (IFPI), Parnaíba 64211-145, PI, Brazil; 2 Interdisciplinary Laboratory of Advanced Materials (LIMAV), Materials Science and Engineering Graduate Program, Federal University of Piauí (UFPI), Teresina 64049-550, PI, Brazil; 3 Department of Chemistry, State University of Londrina, Londrina 86050-482, PR, Brazil; 4 National Institute of Metrology, Quality and Technology (Inmetro), Duque de Caxias 25250-02, RJ, Brazil; 5 Department of Physics, Federal University of Piauí (UFPI), Teresina 64049-550, PI, Brazil; 6 Department of Physics, Federal University of Pernambuco (UFPE), Recife 50670-901, PE, Brazil; 7 Academic Unit of Cabo de Santo Agostinho, Federal Rural University of Pernambuco (UFRPE), Cabo de Santo Agostinho 50670-901, PE, Brazil

**Keywords:** titanate nanotubes, erbium, ion exchange, thermal treatment, optical properties

## Abstract

Titanate nanotubes were synthesized and subjected to an ion exchange reaction with erbium salt aqueous solution to obtain titanate nanotubes exchanged with erbium (3+) ions. In order to evaluate the effects of the thermal treatment atmosphere on the structural and optical properties of erbium titanate nanotubes, we subjected them to heat treatment in air and argon atmospheres. For comparison, titanate nanotubes were also treated in the same conditions. A complete structural and optical characterizations of the samples was performed. The characterizations evidenced the preservation of the morphology with the presence of phases of erbium oxides decorating the surface of the nanotubes. Variations in the dimensions of the samples (diameter and interlamellar space) were promoted by the replacement of Na^+^ by Er^3+^ and the thermal treatment in different atmospheres. In addition, the optical properties were investigated by UV–Vis absorption spectroscopy and photoluminescence spectroscopy. The results revealed that the band gap of the samples depends on the variation of diameter and sodium content caused by ion exchange and thermal treatment. Furthermore, the luminescence strongly depended on vacancies, evidenced mainly by the calcined erbium titanate nanotubes in argon atmosphere. The presence of these vacancies was confirmed by the determination of Urbach energy. The results suggest the use of thermal treated erbium titanate nanotubes in argon atmosphere in optoelectronics and photonics applications, such as photoluminescent devices, displays, and lasers.

## 1. Introduction

Optoelectronic devices use electric charge to generate light, such as via light-emitting diodes (LEDs) and lasers, or use light to generate electric currents, such as via photodetectors and solar cells [1,2,3]. Such devices may be classified into light-sensitive devices and light-generating devices [1]. This classification depends on the physical mechanisms (photoemission, radiative recombination, stimulated emission, photoconductivity, photoelectric effect, among others) responsible for photon generation or the charge used for device operation [2]. In recent years, these devices have been rapidly developing and their efficiency has improved; however, the development of new nanomaterials with multifunctional applications, which are the basis of these devices, is still a growing need [1,3,4].

Semiconductor nanomaterials with one-dimensional structures (such as bars, wires, and tubes) have received much attention in recent years due to their unique physical and chemical properties compared to their respective extended solids. This implies potential applications on new devices [5,6]. These nanomaterials exhibit a wide range of electrical and optical properties that are heavily dependent on shape and size and enable applications in ceramics, gas, sensors, and biosensors, electrochemistry, photocatalysts, nano-piezoelectric and piezoelectric nanogenerators, electromechanical devices, electronics, photonics, and optoelectronics [5,6,7].

Among semiconductor oxides, titanium dioxide nanostructures (TiO_2_) have been extensively investigated due to their promising physical and chemical properties, including low synthesis cost, high specific surface area, non-toxicity, chemical stability, environmental stability, high photoactivity, high bandgap energy, electronic mobility, and conductivity [7,8,9,10]. These properties can be adapted by their structural and surface properties, such as size, morphology, crystallinity, and surface functional groups [11], allowing potential applications in electronics, optoelectronics, magnetoelectronics, optics, catalysts, sensors, and energy conversion [8,12].

Titanate nanotubes (TiNTs), initially proposed by Kasuga et al. [13], stand out among the various nanostructures derived from TiO_2_ [8]. These nanomaterials have interesting physicochemical properties, such as high specific surface area, tunnel-like structure with lamellar walls, low toxicity, and good ion exchange capacity [14,15,16,17,18,19]. In addition, the ion exchange capacity of TiNTs allows surface modifications via interaction with existing OH groups on its surface as well as the insertion of different ions in the interlamellar space [20]. These characteristics allow their use in different types of applications such as capacitor capacitance properties [21], heterogeneous photocatalysis [22], gas sensors [23], solar cells [24], lithium batteries [25], and antibacterial activity [26].

The ion exchange process between TiNTs and other transition metal ions, rare earth elements (RE), and others with various oxidation states, has been widely reported [10,18,19,20,27,28,29,30,31]. Due to its simplicity and high efficiency, this feature offers the opportunity to modify the optical and optoelectronic properties through the introduction and/or intercalation of different metal cations in the crystal structure and/or interlamellar space of the host material [18,30,32].

Some studies have pointed out that TiNTs and other TiO_2_ derivatives are good candidates for RE ion host material in the preparation of photoluminescent materials [4,18,33,34,35,36,37,38,39,40]. The insertion of RE in TiO_2_ nanostructures, including TiNTs, provides optical and luminescent properties that are characterized by sharp excitation and emission bands generated from the electronic transition between 4f levels [39,40,41]. RE insertion can modify bandgap energy and surface properties, improve thermal stability, and create oxygen defects and vacancies that influence the photocatalytic activity of the host material [39,40,42]. In addition, the high fluorescence decay lifetime, strong and sharp emission bands, and a wider absorption band are significant features of laser material [39].

Among several RE, Erbium has been considered one of the most promising due to its high chemical stability, high upconversion luminescence efficiency in the visible and ultraviolet regions, and electronic configuration that provides a long service life in the excited state [43,44,45,46,47]. The insertion of Er^3+^ ions into the host material may also extend the optical absorption to the visible region, decrease the recombination of photoinduced electron–hole pairs, and influence the temperature of the anatase–rutile phase transformation in TiO_2_-based nanostructures [48,49,50].

In addition to the ion exchange process, thermal treatment can be used to improve the physicochemical properties of a material by promoting its crystallinity, converting it into a more crystalline phase, or reducing surface defects [51]. TiNTs thermal treatment invariably leads to crystalline TiO_2_ formation with the achieved phase being determined by the temperature treatment and potentially by the original structure [51]. Moreover, the physicochemical properties of TiNTs (crystallinity, structure, stoichiometry, surface chemistry, among others) are strongly dependent on thermal treatment conditions (time, heating speed, atmosphere, etc.) [11]. Recently, one of the topics that has been frequently discussed is the effect of the thermal treatment atmosphere on different nanostructures [52,53,54,55,56].

Here, we synthesized TiNTs (NaTiNTs and ErTiNTs) using the microwave-assisted alkaline hydrothermal method, subjected to thermal treatment in air and inert atmospheres. We focus on a more detailed characterization and the influence of thermal treatment atmosphere on the morphology, microstructure, and optical (electronic) properties of titanate nanotubes.

## 2. Materials and Methods

### 2.1. Preparation, Ion Exchange Reaction and Thermal Treatment of Titanate Nanotubes

Sodium titanate nanotubes (NaTiNTs) were synthesized based on the previous work [57]. In this synthesis, 3.0 g of TiO_2_ anatase (99.98%, Sigma-Aldrich, Jurutaba, Brazil) was dispersed in 90 mL of NaOH aqueous solution (98%, Dinamica, Indaiatuba, Brazil) to a concentration of 10 Mol L^−1^and magnetically stirred for 30 min. The solution was then dropped in a Teflon^®^ reactor, autoclaved and subjected to microwave irradiation in an adapted house-microwave oven at 160 °C for 4 h. The obtained solid was centrifuged at 3500 rpm, washed with deionized water to pH = 10, and dried under vacuum for 24 h [57]. Then, the prepared NaTiNTs were subjected to ion exchange reaction with metal ions (Er^3+^) at room temperature without any additional protonation process. For this, 500 mg of NaTiNTs were suspended in 500 mL at 0.01 Mol L^−1^ of an aqueous solution of [Er (H_2_O)_5_] (NO_3_)_3_ (99.9%, Sigma-Aldrich) (pH ≈ 5) followed by magnetic agitation at ambient temperature for 24 h. The solid was isolated by centrifugation at 3000 rpm and washed several times with deionized water for removal of nitrates. Afterward, samples exchanged with ions Er (ErTiNTs) were vacuum dried for 24 h. Finally, 200 mg of NaTiNTs and ErTiNTs were inserted in an alumina crucible, which was then placed into a quartz tube and taken to a tubular furnace for thermal treatment. The samples were heated with temperatures of 200, 400, 600, and 800 °C for 2 h with ramp heating of 10 °C/min, in air and inert (Ar) atmospheres.

### 2.2. Characterizations

Raman spectroscopy experiments were performed using a Bruker Senterra Raman Spectrometer equipped with an Olympus BX51 microscope, a charge-coupled device detector, 20X focus lens and a laser operating at 785 nm. The spectra were obtained from three accumulations of 20 s with an output laser power of 25 mW and a resolution of 4 cm^−1^ in the region between 1200 cm^−1^ and 85 cm^−1^. X-ray powder diffraction patterns were obtained using a Shimadzu XRD 6000 diffractometer employing Cu-Κα radiation (λ = 1.54 Å). Data were collected with a scanning speed of 1°/min in a range of 5–60° (2θ). Scanning electron microscopy (SEM) and energy dispersive X-ray spectroscopy (EDS) was performed with a model FEI Quanta 250 FEG microscope with a Genesis Apollo X SSD detector from EDAX. High-resolution transmission electron microscopy (HRTEM) images and scanning transmission electron microscopy (STEM) images of ErTiNTs and ErTiNTs samples heat treated at 400 °C in argon atmosphere were acquired using a probe-corrected Titan 80–300 kV (FEI Co.) instrument working at 300 kV. STEM images were acquired with a high-angle annular dark field detector (HAADF) at a distance of 100 mm, which improves the contrast between Ti and Er. These samples were prepared by inserting an aqueous suspension of the material powder into a carbon-coated copper grid to allow water to evaporate at room temperature. The transmission electron microscope (TEM) Tecnai G2-20-FEI Supertwin 200 kV (FEI, Hillsboro, OR, USA) was used to acquire TEM images of ErTiNT samples heat treated in an argon atmosphere at 600 and 800 ℃ and ErTiNTs samples heat treated in an air atmosphere at 400, 600, and 800 ℃. These samples were prepared by suspending the powder of the material in water and placing it on a copper grid coated with carbon. Diffuse solid-state UV–Vis reflectance spectroscopy was performed in a UV-2600 Shimadzu spectrophotometer (Shimadzu, Kyoto, Japan) in absorbance mode in the wavelength in the range of 200 to 800 nm. The energy of the optical band gap was calculated using the Kubelka–Munk method for an indirect transition according to the relevant theoretical considerations [58]. The photoluminescence spectroscopy (PL) experiments were performed in a Horiba Jobin Yvon Fluorolog-3 spectrofluorometer equipped with double excitation and emission monochromator (FL-1039/40) (Horiba, Piscataway, NJ, USA). The emission spectra were obtained under 370 nm excitation and the excitation spectra were acquired by monitoring the emission at 430 nm. All measurements were collected at room temperature.

## 3. Results

### 3.1. Raman Spectroscopy

**Figure 1:**
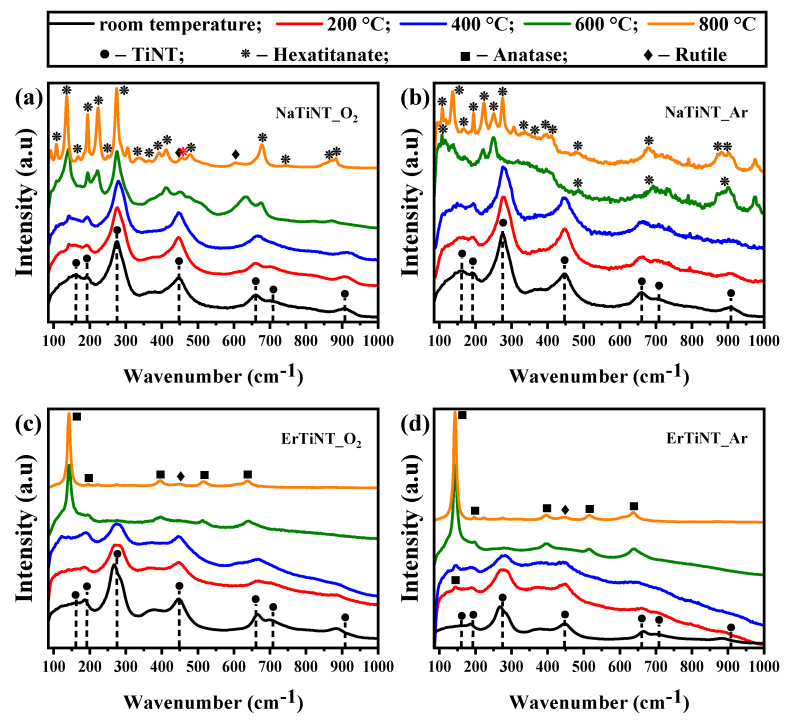
Raman spectra of NaTiNTs and ErTiNTs heat treated in air, (**a**) and (**c**), respectively, and argon (Ar) atmosphere, (**b**) and (**d**), respectively. The dashed lines show the characteristic peaks of the TiNTs, and the symbols indicate the phases observed during thermal treatment at different temperatures. The upper caption is the same for all samples.

Figure 1 shows the Raman spectrum of NaTiNTs and ErTiNTs treated in air and argon atmospheres. NaTiNTs exhibit modes around 160, 190, 275, 446, 657, 703, and 906 cm^−1^ characteristic of titanate nanotubes [12,58]. The modes located at 160 and 190 cm^−1^ are related to Na-O-Ti stretching of the lattice [19], and the modes 275, 446, 657, and 703 cm^−1^ correspond to the framework Ti-O-Ti vibrations [59]. The mode at 275 cm^−1^ has been reported as an inherent mode of NaTiNTs, namely a Ti-O vibration affected by a nearby Na^+^ ion [12]. Furthermore, the mode located at 906 cm^−1^ is related to Ti-O bonds, whose oxygen atom is not shared between TiO_6_ units [19,26,57]. With increased temperature, the modes related to the TiNT structure of the NaTiNTs samples (Figure 1a,b) are preserved up to 400 °C in both atmospheres. In Figure 1a, beginning at 600 °C, NaTiNTs calcined in air atmosphere (NaTiNT_O_2_) transform into the sodium hexatitanate phase (Na_2_Ti_6_O_13_) which is characterized by a tunnel-like structure, without the presence of layers [57,60]. This transformation is attributed to the emergence of modes around 107, 167, 194, 222, 249, 275, 333, 363, 411, 455, 479, 677, 741, and 870 cm^−1^ [61,62]. We also observed low-intensity peaks around 450 and 605 cm^−1^ related to the rutile phase between 600 and 800 °C [42,57,61,63,64]. In Figure 1b, NaTiNTs treated in argon atmosphere (NaTiNT_Ar) underwent the same transformation as NaTiNT_O_2_ from 600 °C; however, the peaks located at 194, 275, and 677 cm^−1^ are not observed as well as the peaks related to the rutile phase.

In ErTiNTs (Figure 1c,d), the ion exchange with Er^3+^ caused changes in the intensity and wavenumber of the modes located in 275, 657, 703, and 906 cm^−1^ [5,18,29,57,65,66]. These changes indicate that the ion exchange with Er^3+^—and, consequently, the reduction of Na^+^ content in the TiNTs structure—modifies the energy of these vibrations due to the difference in atomic mass and ionic radius of Na and Er (22.99 amu and 0.97 Å for Na^+^ and 167.26 amu and 1.76 Å for Er^3+^) [61,67,68,69,70]. With the increase in temperature, ErTiNTs behaved the same in both atmospheres, which includes the formation of the rutile phase evidenced by the mode at 444 cm^−1^ and the presence of the modes referring to the anatase phase [42,57]. Furthermore, no additional peaks related to erbium oxide are observed, indicating the absence of separate phases due to ion exchange and/or thermal treatment independent of atmosphere [45,57].

### 3.2. X-ray Diffraction

**Figure 2:**
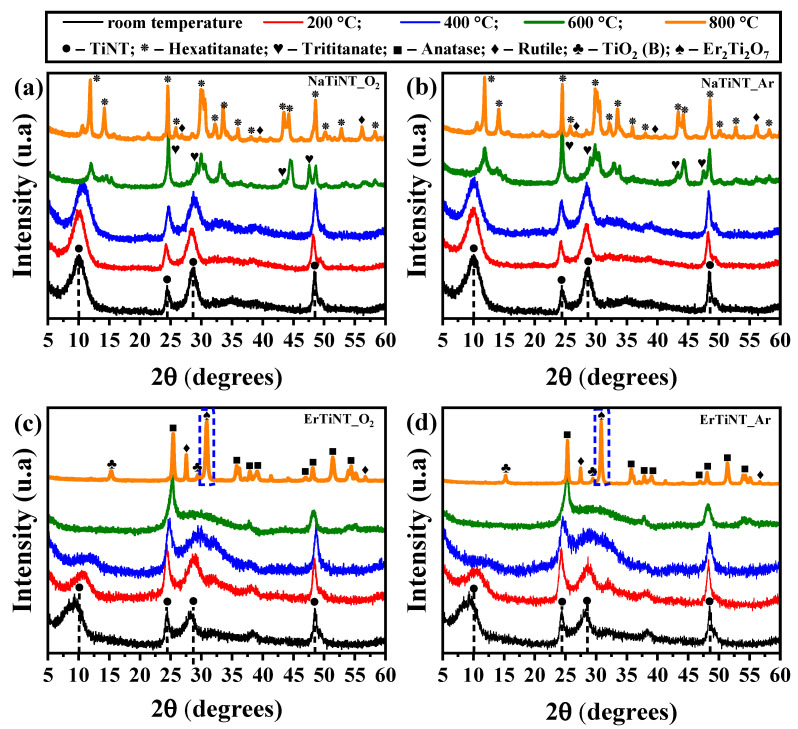
XRD diffractograms of NaTiNTs and ErTiNTs heat treated in air (O_2_), (**a**) and (**c**), respectively, and argon atmospheres, (**b**) and (**d**), respectively. The dashed lines show the characteristic peaks of the TiNTs, and the symbols indicate the phases observed during thermal treatment at different temperatures. The dashed rectangles highlight the peak related to the erbium oxide formed. The upper caption is the same for all samples.

The X-ray diffractograms of the NaTiNTs and ErTiNTs are provided in Figure 2. The effects of curvature on TiNT structure are evidenced by broad reflections in all patterns analyzed. The asymmetric increase in the peaks in relation to bulk titanates is due to the distortion of the unit cell caused by the curvature of the TiNT structure [71,72]. In NaTiNTs, the characteristic crystallographic planes (200), (110), (211), and (020) of TiNTs are characterized by the peaks located at 2θ = 10, 24, 28, and 48°, respectively [73,74,75]. These peaks can be associated with the crystallographic data of the Na_2_Ti_3_O_7_⋅nH_2_O phase (JCPDS card n° 13-3129) [19]. During thermal treatment, NaTiNT_O_2_ (Figure 2a) and NaTiNT_Ar (Figure 2b) preserve the TiNT structure up to 400 °C, as observed in Raman spectroscopy experiments. We also observe the formation of the sodium hexatitanate phase at 600 °C. This transition is complete at 800 °C, evidenced by the peaks located around 11.9; 14.1; 24.5; 25.8; 29.9; 32.2; 33.5; 35.9; 38.1; 43.4; 44.2; and 48.6° (2θ) [68,76]. We also observed the presence of peaks around 27.7 and 39.5° (2θ) referring to the rutile phase [42]. We can also suggest that, regardless of the atmosphere, the heat treatment promotes a greater crystallinity of the samples. This statement is supported by the higher intensity of the peaks observed in relation to the background [68].

The XRD profile of ErTiNTs (Figure 2c,d) indicates that both the structure and morphology of the TiNT were preserved after insertion of Er^3+^ ions into the interlamellar space. The peak located around 10° (plane (200)) related to the interlamellar distance shows a slight shift to the lower 2θ position. This displacement can be associated with an increase in the distance between the layers of the TiNT structure caused by ion exchange with Er^3+^ ions. This increase was expected due to the difference between the ionic rays (sodium less than erbium) [18]. Moreover, this change in the distance between the layers increases the curvature of the TiNT structure, causing a large distortion in the unitary cell [18,57,72]. Changes in planes 24 and 28° (2θ) (diagonal planes) may be related to the rearrangement of Er^3+^ ions in the layered titanate at different coordination positions when compared to NaTiNTs [18,65,74]. With the heat treatment, the samples of ErTiNTs in the same way that NaTiNTs maintained the TiNT structure up to 400 °C. At 600 °C, the formation of the anatase phase is evidenced by the emergence of modes around 2θ = 24.4; 37.9; 48.1; 54.0; and 55.1° [58,77]. The presence of other TiO_2_ polymorphs, including TiO_2_ phase (B) and rutile (27.7°), with low intensity in 800 °C [61,78], as well as the formation, in both atmospheres, of Er_2_Ti_2_O_7_ (JCPDS card n°. 18-0499), is evidenced by the peak located around 31.0° [79].

### 3.3. Morphology and Composition Analysis

The analyses performed by Raman spectroscopy and XRD indicated changes in NaTiNTs after ion exchange with Er^3+^ ions due to substitution of Na^+^ by erbium ions in the interlamellar space. The chemical composition of NaTiNTs and ErTiNTs was investigated by EDS to quantify the substitution of Na^+^ by erbium ions. Atomic ratios Na/Ti and Er/Ti are provided in Table 1. We can observe that the amount of Na^+^ is higher in NaTiNTs compared to ErTiNTs, suggesting that the ion exchange reaction with ions Er^3+^ promotes a decrease in Na^+^ allowing to observe the presence of ions Er^3+^. We also observe the presence of residual Na^+^ in the ErTiNTs. This indicates that ion exchange is efficient, but partially [57]. In addition, the thermal treatment, independent of the atmosphere, did not influence the changes in the Na/Ti or Er/Ti ratios, indicating dependence only with the ion exchange step.

**Figure 3:**
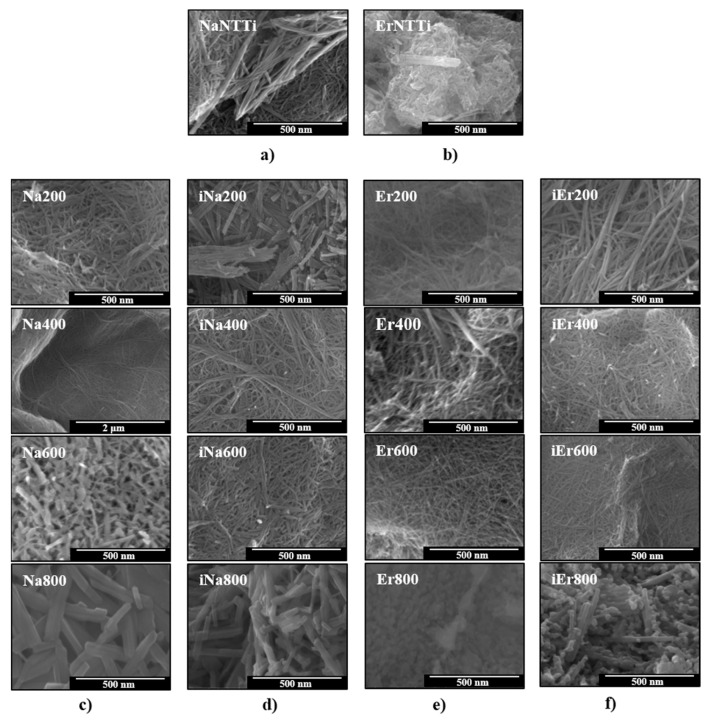
SEM images of (**a**) NaTiNTs and (**b**) ErTiNTs heat treated in air and argon atmosphere. In (**c**,**d**), the fibrillar morphology of NaTiNTs is preserved with increasing temperature but with a different aspect in 800 °C, indicating the probable formation of hexatitanate nanorods (Na_2_Ti_6_O_13_). In (**e**,**f**), the fibrillar morphology of ErTiNTs is preserved up to 600 °C and clusters of NPs anatase are formed at 800 °C. The prefix “i” in columns (**d**,**f**) indicates the samples treated in inert atmosphere (argon).

Figure 3 (SEM images) illustrates fibrillar (1D) morphology of the NaTiNTs and ErTiNTs, and careful observation of the NaTiNT and ErTiNT samples reveal that TiNTs are connected in bundles [18]. From 600 ℃, the heat treatment of NaTiNTs (Figure 3c,d) leads to the formation of Na_2_Ti_6_O_13_, which presents a rod-like morphology (monoclinic phase), independent of the atmosphere [76]. The ion exchange did not alter the TiNTs fibrillar morphology of the ErTiNTs, which was maintained up to 600 °C (also independent of the atmosphere), indicating better thermal stability compared to TiNTs exchanged ionically with other RE [57]. The formation of the anatase phase at 800 °C is corroborated by the presence of the morphology composed of nanoparticles, not nanorods (Figure 3e,f) as in the precursor sample [57].

**Figure 4:**
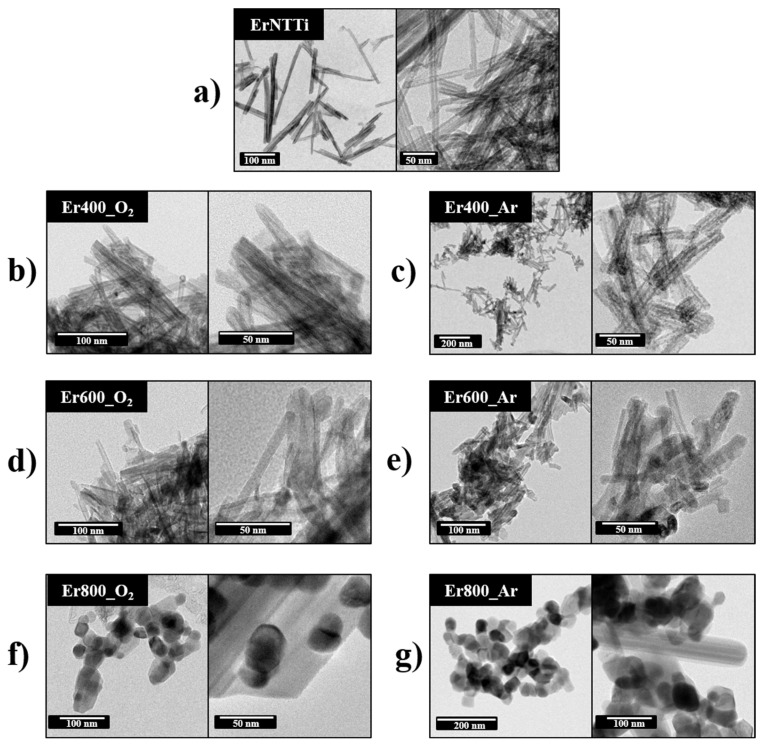
TEM images of ErTiNT without treatment (**a**), ErTiNT treated in air, 400 °C (**b**), 600 °C (**d**), 800 °C (**f**), and treated in Argon, 400 °C (**c**), 600 °C (**e**), 800 °C (**g**), showing the tubular morphology until 400 °C and the collapse of tubes and formation of NPs clusters of TiO_2_ anatase.

Except for the analysis performed by XRD, the other techniques were not conclusive regarding the formation of nanoparticles (NPs) of erbium oxides in ErTiNTs. Thus, we performed TEM studies to confirm the results obtained by XRD. Figure 4 shows the TEM analysis of ErTiNTs at room temperature and calcined from 400 °C in air and argon atmospheres. Figure 4a illustrates that the tubular morphology of TiNTs was preserved after ion exchange, corroborating with the Raman and XRD results. This morphology was preserved up to 400 ℃ and, from 600 ℃, the nanotubes collapse into pieces of shorter lengths and begin to form clusters of NPs. At 800 °C, we observed the complete formation of clusters of NPs.

**Figure 5:**
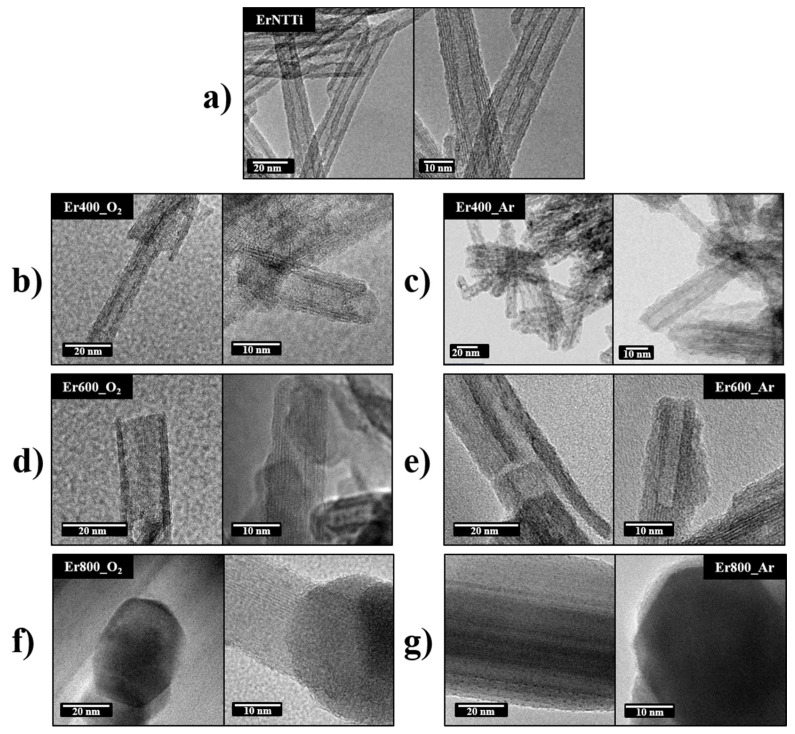
TEM and HAADF-STEM images of ErTNT without treatment (**a**), ErTiNT treated in air, 400 °C (**b**), 600 °C (**d**), 800 °C (**f**), and treated in Argon, 400 °C (**c**), 600 °C (**e**), 800 °C (**g**), showing Er-based NPs and NPs of TiO_2_ anatase on the surface of titanate nanotubes.

Figure 5 shows the HRTEM images of the calcined ErTiNTs in air and inert atmosphere. ErTiNTs (Figure 5a–c) have an average length of 190 nm, an external diameter around 10 nm, and an average internal diameter of 4 nm. In addition, the interlamellar distance is around 0.82 nm [18]; for NaTiNTs, this measurement is around 0.70 nm in accordance with previous works [58]. These dimensions are preserved up to 400 °C in both atmospheres. However, beginning at 600 °C, we observe reductions in external (<10 nm) and internal (<3.5 nm) diameters and interlamellar distance (<0.7 nm). This behavior can be attributed to the dehydration process of the OH interlamellar group during the heat treatment, independent of the atmosphere. In addition, the clusters of NPs formed have crystalline NPs with interplanar distances around 0.33 nm, consistent with NPs of TiO_2_ anatase, which agrees with previous studies [14] and corroborates the phase transition observed by Raman and XRD measurements.

**Figure 6:**
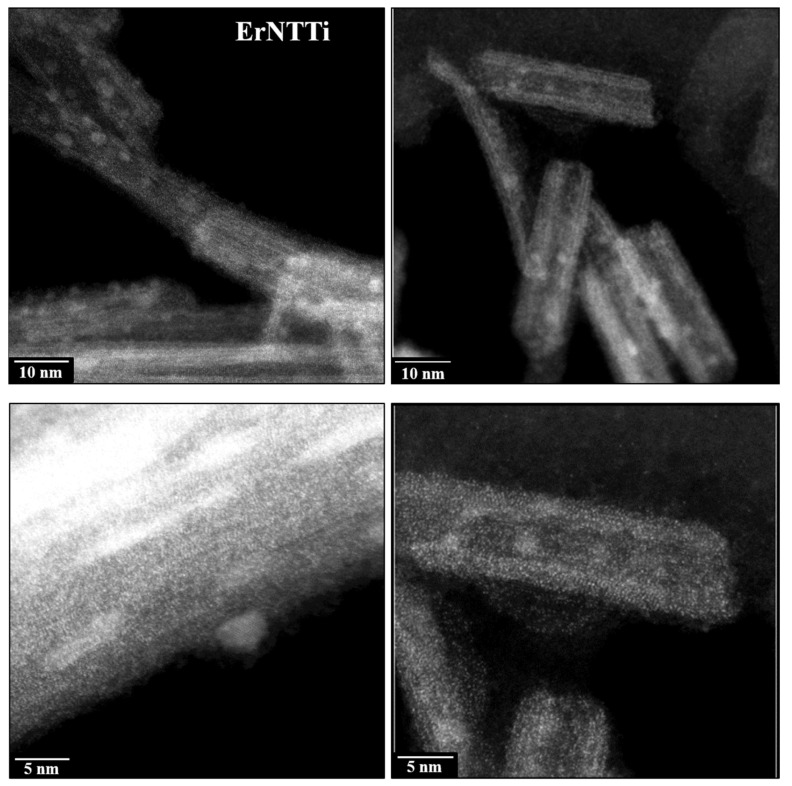
HAADF-STEM images of ErTiNT showing Er-based NPs and NPs of TiO_2_ anatase on the surface of titanate nanotubes. The two NPS are lighter than TiNTs; however, Er-based NPs are smaller and have higher brightness.

In addition to TEM analyses, HAADF-STEM images of ErTiNTs show two different types of NPs (Figure 6). The first type presents very small NPs, which are probably erbium oxide clusters made of few atoms since they exhibit high Z contrast when compared to TiNTs. It is known that the contrast Z of the HAADF-STEM image is linked to the different atomic numbers of the sample atoms (22 for Ti and 68 for Er). These very small Er-based NPs are evenly distributed in TiNTs. The second type of NPs, with an average size of 5 nm, are brighter than TiNTs but darker than NPs based on Er. These two facts suggest that these NPs are in the TiO_2_ structure. Probably, TiNTs are decorated with TiO_2_ NPs in the anatase phase as observed in our previously works [20,58] about the synthesis method used here. In general, the morphological and structural properties of NaTiNTs and ErTiNTs showed strong dependence on the temperature; however, dependence on the different atmospheres were not observed.

### 3.4. Optical Properties

**Figure 7:**
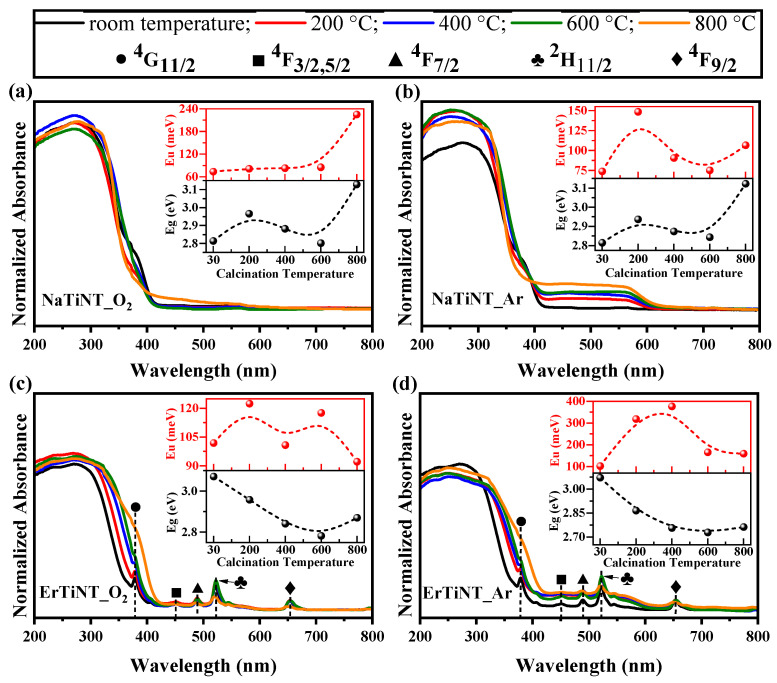
UV–Vis absorption spectrum of NaTiNTs heat treated in air (**a**) and Argon atmosphere (**b**), and ErTiNTs heat treated in air and argon atmospheres, (**c**) and (**d**), respectively. The dashed lines in the ErTiNTs spectra show absorption peaks that change with increasing temperature. The symbols on the absorption peaks indicate the electronic transitions from the ground state ^2^I_15/2_. The inserts in the graphs show the band gap and Urbach energy variations of the samples with increasing temperature. The upper caption is the same for all samples.

In order to evaluate the influence of different calcination atmosphere on the optical properties of the samples, UV–Vis absorption spectroscopy and photoluminescence (PL) experiments were performed. The UV–Visible absorption spectroscopy (solid) was performed on NaTiNTs and ErTiNTs to obtain the optical spectrum (Figure 7). We can observe that NaTiNTs have a strong absorption edge in the ultraviolet region (below 400 nm), associated with excitation of the electron O2p from the valence band to the Ti3d level of the conduction band [18,77]. With increasing temperature, NaTiNT_O_2_ (Figure 7a) presented a narrow absorption edge varying between 365 and 410 nm at 800 °C [57,75,80]. NaTiNT_Ar (Figure 7b) showed a weak broad absorption range in the visible region extending to 630 nm. It is known that the electronic band gap of TiNTs depends on the tubular diameter [11]. Thus, the redshift observed in the absorption of NaTiNT_Ar may be related to the dehydration process of the OH interlamellar group, which implies contraction and, consequently, reduced diameter of the TiNT structure, less electronic band gap, and better light absorption in the visible region [11,81,82]. In addition, the dehydration process of TiNTs during thermal treatment generates oxygen vacancies that can be recombination centers of photogenerated electrons/holes, influenced by the optical absorption of the sample [57,81]. The main difference between the optical absorptions of the samples must be in the reduction of oxygen vacancies by oxidation reactions in NaTiNT_O_2_ (with a large amount of oxygen) and the maintenance of these vacancies in NaTiNT_Ar, as oxidation reactions are reduced in inert atmospheres.

The optical absorption spectrum of ErTiNTs presents, in addition to the absorption band in the UV region, characteristic of the TiNTs structures, a peak at 379 nm referring to the transition ^4^I_15/2_ → ^4^G_11/2_ of the Er^3+^ ion [83]. Peaks in the visible region are also observed around 451, 489, 524, and 655 nm and can be assigned to the transition from ground state ^2^I_15/2_ to excited states ^4^F_3/2,5/2_, ^4^F_7/2_, ^2^H_11/2_, and ^4^F_9/2_ of the Er^3+^ ions [44,50,57,84,85]. When the temperature was increased, ErTiNT_O_2_ keeps its spectral profile practically unchanged. The only changes observed are related to the peaks located at 379 nm, which reduces its intensity at 600 °C and disappears at 800 °C, and at 534 nm, which has increased intensity at 600 °C followed by a decrease at 800 °C. In addition, a redshift is observed compared to NaTiNT_O_2_. Thermal treatment displaced the absorption edge of ErTiNT_O_2_, which corresponds to a change in the band gap that may arise due to the phase transformations suffered by the sample [86]. The difference in the behavior of NaTiNT_O_2_ and ErTiNT_O_2_ spectra for the same thermal treatment atmosphere may be associated with structural dehydration caused by ion exchange and, consequently, the reduction of the amount of sodium in the sample [86,87] and the formation of different phases during thermal treatment. [57,86,87]. NaTiNTs changed to the hexatitanate phase (Na_2_Ti_6_O_13_), which has a higher band gap than TiO_2_ anatase resulting from the transition from the TiNT phase of ErTiNTs [57,88]. The behavior of ErTiNT_Ar was similar to NaTiNT_Ar for the mechanisms that influence the improvement of optical absorption. In addition, ErTiNT_Ar display the same behavior at the peaks related to Er^3+^ ions observed in the UV and visible region of ErTiNT_O_2_, which leads to synergistic effects between inert atmosphere thermal treatment and ion exchange with Er^3+^ ions, tuning the optical properties of ErTiNT_Ar compared to unmodified NaTiNTs.

To analyze in detail the influence of ion exchange and heat treatment on the optical absorption behavior of the samples, we estimated the band gap and Urbach energy (see insets in Figure 7 and Supplementary Figures S1–S4 in Supplementary Material) of NaTiNTs and ErTiNTs at different treatment temperatures and atmospheres. The indirect band gap of TiNT was estimated using the Tauc plot by plotting ${\left(\alpha h\nu \right)}^{1/2}$ versus hν [30]. From the extrapolation of the linear part of the graph, the gap is the intersection with the energy axis (hν). We also calculated the Urbach energy, which is associated with the absorption tail produced by the defects located inside the gap. The Urbach equation is given by $\alpha =\alpha_{0}\exp\left(E/E_{u}\right)$, where α is the absorption coefficient, E is the photon energy, and $E_{u}$ is the Urbach energy [89]. The Urbach energy is obtained by plotting $Ln\;\left(\alpha \right)$ versus E, where the value of $E_{u}$ is given by the reciprocal slope of the linear portion, below the optical gap. The band gap values ($E_{g})$ for NaTiNT and ErTiNT are 2.81 eV and 3.07 eV, respectively. The $E_{g}$ value obtained for the NaTiNT sample of 2.81eV is much lower than the values reported for TiNTs, which are generally above 3 eV [19,20,90,91,92], shifting its absorption edge to the almost visible region. The value obtained is similar to the result reported by Bem et al. [93], who synthesized TiNTs with different sodium contents using a hydrothermal approach. Wang et al. [94] found a similar value for TiO_2_ nanotubes, which was associated with the sodium content of the samples. This implies that sodium content influences the band gap of TiO_2_-based materials. On the other hand, ErTiNTs presented higher band gap than NaTiNTs at room temperature. It is known that the sodium content decreased in the ionically exchange sample, which should produce lower band gap values; however, as the insertion of Er^3+^ increases the interlamellar distance and consequently the diameter of the tube, the band gap of the ErTiNTs is larger. The tubular diameter probably has greater influence than the sodium content in the band gap of TiNTs.

The band gap of NaTiNTs suffered an increase at 200 °C, followed by a decrease at 400 and 600 °C and a new increase at 800 °C. The behavior of the band gap at 200 °C was opposite to those found in the literature, where the expected value would be lower than the uncalcined sample [87,95]. This anomalous behavior can be attributed to the growth of the external diameter and reduction of the internal diameter of the TiNTs at low thermal treatment temperatures, similar to the behavior observed by Zhang et al. [96]. This increase in diameter directly influences the band gap of TiO_2_-based materials [11,97]. Between 400 and 600 °C, the dehydration process of the interlamellar OH groups, controlled by the presence of sodium [87], promoted the reduction of both the interlamellar space and TiNTs diameter, reducing the band gap. The presence of sodium influenced the structural transformation of TiNT to anatase through the slower dehydration process, shifting the phase transition to higher temperatures [87,98]. Thus, NaTiNTs with Na_2_Ti_3_O_7_ structure evolved to the hexatitanate phase (Na_2_Ti_6_O_13_) at 800 °C. The basic difference between these structures is that the first presented a lamellar structure with corrugated layers of Ti_3_O_7_^2−^ and two interlamellar Na^+^ ions, while the second had a tunnel-like structure with two Na^+^ ions inside the tunnel. This structure could originate from the sharing of Ti_3_O_7_^2−^ units between adjacent Na_2_Ti_3_O_7_ layers [99]. This phase has a larger band gap than TiNTs [57,88]. ErTiNTs presented a reduction in the band gap with increasing temperature up to 600 °C, followed by an increase by 800 °C due to the transition of the TiNT phase to anatase phase. However, the value of the band gap was lower than that of the NaTiNTs that changed to the hexatitanate phase as mentioned above. In addition, the values obtained for Urbach energy suggest an increase in the number of oxygen vacancies promoted by thermal treatment and ion exchange. These oxygen vacancies disturbed the band structure of the samples, promoting the observed variations of the band gaps [100].

**Figure 8:**
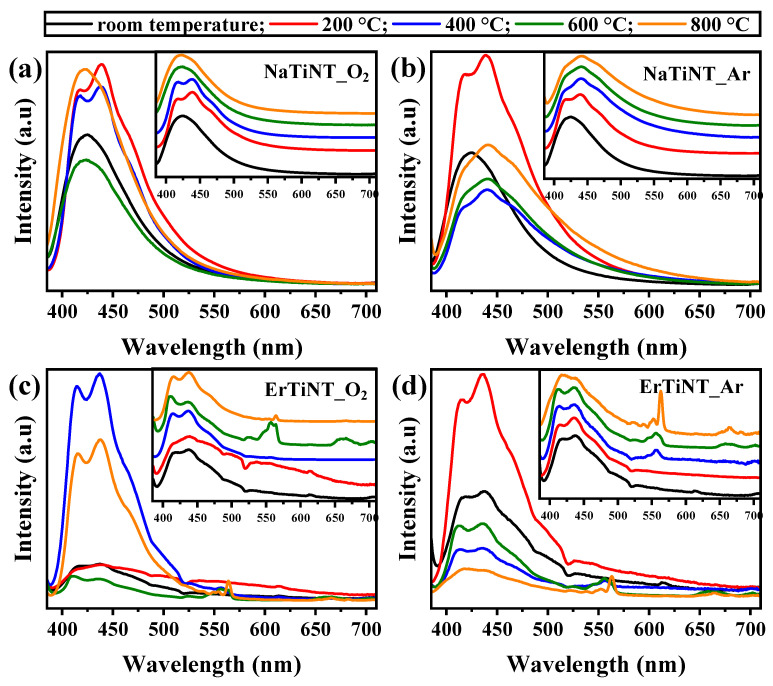
Emission spectra PL (excitation at 370 nm) NaTiNTs heat treated in air (**a**) and Argon atmosphere (**b**), and ErTiNTs heat treated in air and argon atmospheres, (**c**) and (**d**), respectively. Variations in PL intensity occur with increasing temperature in all samples. The insets show the spectral profile of the samples normalized and translated vertically for ease of visualization.

**Figure 9:**
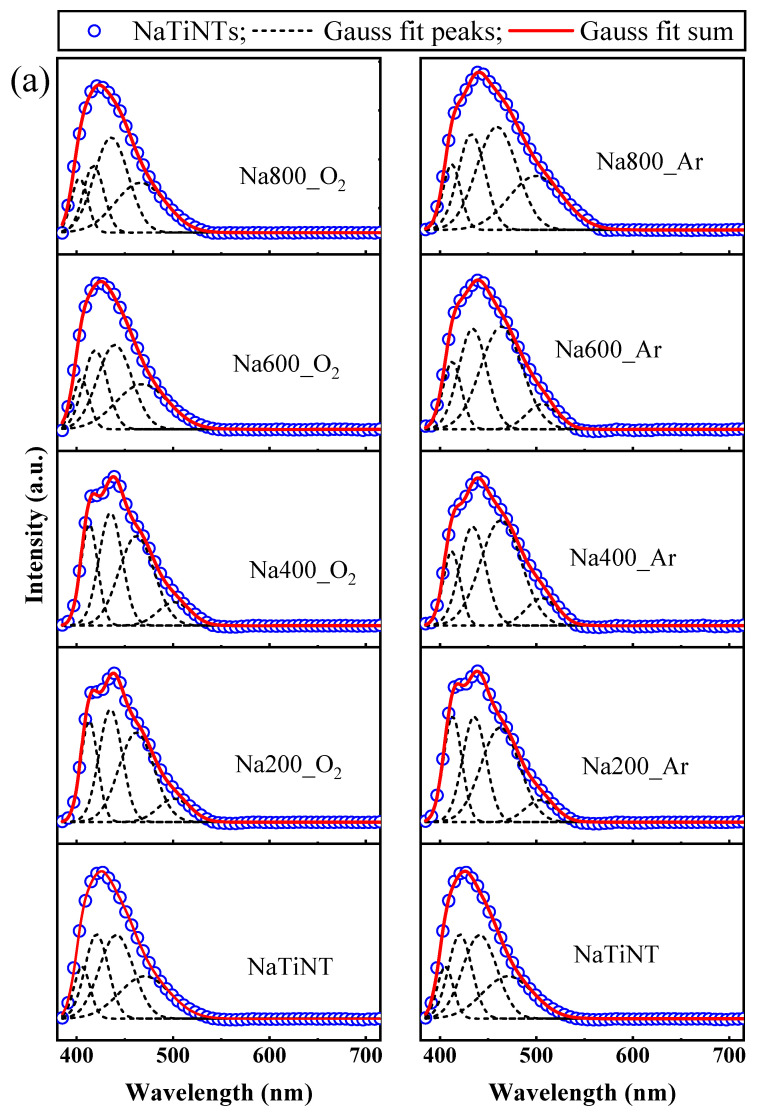

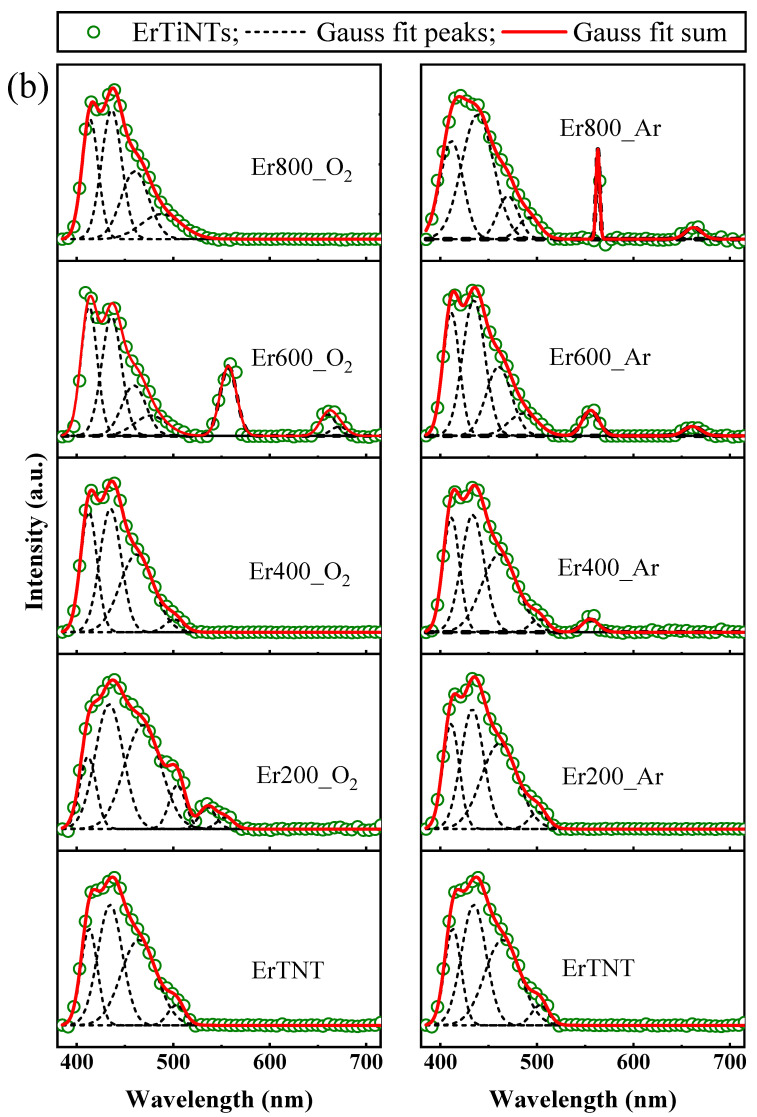
(**a**) Deconvolution of the PL emission spectrum (excitation at 370 nm) of NaTiNTs heat treated in air and argon atmosphere (the upper caption is the same for all samples); (**b**) deconvolution of the PL emission spectrum (excitation at 370 nm) of ErTiNTs heat treated in air and argon atmosphere (the upper caption is the same for all samples).

The recombination rate of electron–hole photogenerated pairs in TiO_2_-based materials is usually studied by photoluminescence spectroscopy (PL), where the PL intensity indicates the ability to stabilize photogenerated excitons [101,102,103]. At low-intensity, PL spectrum reflects a lower rate of recombination of the photogenerated excitons, for example [57,103]. In the case of TiO_2_-based materials, PL signals are mainly due to oxygen vacancies, surface defects, and self-trapped excitons [57,104], while Er^3+^ ions have characteristic lines with narrow and sharp emission bands and a specific wavelength, originating from f-f transitions in the 4f orbital [57,105]. In Figure 8, it is possible to observe that the profiles of the PL emission spectra of NaTiNTs and ErTiNTs are similar. They have a center of band located around 425 nm. This band consists of four peaks located around 405, 421, 442, and 474 nm (Figure 9) that are originated from the recombination of the electron–hole pairs in the TNT structure after photoactivation [57,106,107]. The excitation spectra of NaTiNTs and ErTiNTs acquired by emission monitoring at 430 nm (Supplementary Figure S5 in Supplementary Material) exhibit a wide band with maximum intensity around 370 nm. This band has been attributed to titanate structures [57]. In addition, ErTiNTs have a reduction in band intensity compared to NaTiNTs and an absence of peaks characteristic of Er^3+^ ions, indicating that the emission arises from the indirect excitation of the Er^3+^ ions from the energy transfer of the excitons generated in the host TiNT. In other words, the TiNTs network absorbs the excitation energy of UV light via transition between bands. This energy is then transferred to the Er^3+^ ions. This suggests that these ions are inserted into the spaces between the layers of the TiNT structure [45,57].

NaTiNT_O_2_ (Figure 8a) displays an increase in intensity up to 200 °C, a decrease between 400 °C and 600 °C, and, finally, a new increase in intensity at 800 °C. This PL intensity increase at 800 °C was discussed in our previous study [57], which attributed it to the dehydration of OH groups present between the layers of the TiNT structure. This dehydration promotes the contraction and collapse of the nanotubular structure [81] and generated oxygen vacancies that can act as recombination centers for the photogenerated electron–hole pairs, resulting in a more intense PL signal [57,81]. NaTiNT_Ar (Figure 8b) had different behavior in PL intensity variations. At 200 °C, the PL intensity increased, decreasing dramatically by 400 °C, followed by further increases by 600 and 800 ℃. However, the intensity at 800 °C approached the intensity of the uncalcined NaTiNTs, different from the high intensity observed in the NaTiNT_O_2_ spectrum. A lower intensity of PL indicates a lower rate of recombination of the electron–hole pair and, consequently, a smaller band gap, in addition to a greater number of photogenerated carriers with longer lifetimes [30,57]. This greater separation of the electron–hole pair is improved due to the greater number of defects and vacancies [30] generated during thermal treatment in an inert atmosphere, resulting in improved photocatalytic properties and photovoltaic efficiency of the sample [57,107,108].

The PL spectrum of ErTiNT_O_2_ is presented in Figure 8c, where we observed an increase in intensity up to 400 °C, followed by a decrease in 600 °C, and a new increase between 600 and 800 °C. This increased intensity by 800 °C is the result of greater recombination of the charge carriers. [57]. This higher recombination is promoted by oxygen vacancies generated by thermal treatment in the air atmosphere and by the insertion of Er^3+^ ions in host TiNT [57,109,110]. The presence of peaks located between 550 and 565 nm (visible region) refers to the transitions of the excited states (^4^S_3/2_) of the ground state (^4^I_15/2_) of the Er^3+^ ions generated from the indirect excitation of Er^3+^ ions, as discussed previously [45]. These emissions are also attributed to the strong ion–phonon interaction, where the phonon energy is small in TiO_2_-based hosts, while Er^3+^ ions have higher energy due to the 4f→5d configuration implying a larger gap, making the emission of certain energy levels more probable than multi-phonon relaxation. [111]. The emergence of a peak around 665 nm at 600 °C refers to the transition ^4^F_9/2_ → ^4^I_15/2_ [111,112], whose intensity decreases considerably by 800 °C. This behavior has already been reported for Er^3+^ ions hosted in TiO_2_-based materials, where the PL intensity of emissions in the visible spectrum first grows with increasing temperature and then decreases [112]. In Figure 8d, ErTiNT_Ar shows the growth of intensity PL at 200 °C, a reduction at 400 °C, followed by a small increase at 600 °C and a new reduction at 800 °C, which presents the lowest intensity among the thermal treatment temperatures used. These variations are similar to those observed in NaTiNT_Ar; however, with a lower intensity at 800 °C, corroborating with the previously discussed behavior. The greater separation of excitons—that is, the lower recombination rate—is improved due to the greater number of defects and vacancies generated during thermal treatment in an inert atmosphere and an increased lifetime of the photogenerated carriers’ that can engage in photochemical reactions, improving the photocatalytic properties of the sample [113]. In addition, when the sample interacts with light, some well-defined energy levels are excited and then decay; however, this decay does not necessarily occur radiatively, which may explain the weaker PL intensity. This decay consists of an sequence of energy transfer of phonons from the TiNT network, combined with a low radiative energy process that ends up being seen as a PL signal [114].

An analysis of the position of the peaks in bands referring to the TiNT structure was performed with Gaussian function fitting (see Figure 9 and Figure S6 in Supplementary Material). As the peaks referring to the Er^3+^ ions did not change their positions, we will not discuss them here. With a temperature rise up to 400 °C, NaTiNT_O_2_ and NaTiNT_Ar exhibited a gradual redshift that can be attributed to the thermal occupancy of the higher vibronic levels [115]. However, between 400 and 600 °C, NaTiNT_O_2_ had an abrupt blueshift, which is reduced between 600 and 800 °C; while in NaTiNT_Ar, this blueshift started to reduce at 400 °C. This blueshift can be attributed to the collapse of the TiNT structure that is converted for the hexatitanate phase (nanorods), which can lead to a quantum size effect [116]. ErTiNT_O_2_ and ErTiNT_Ar exhibited a redshift in PL with the insertion of Er^3+^ ions. This redshift of the band gap has been observed in previous works and can be attributed to the emission of PL by vacancies of oxygen or defects located between the bands O2p and the bands 4f of RE in NPs of RE oxides formed on the surface of nanotubular titanates, as shown by TEM images [19,31,57,117,118,119]. With increased temperature, ErTiNT_O_2_ presented a slight blueshift while ErTiNT_Ar maintained the position of the peaks almost constantly. This behavior could be associated with the transition from the TiNT phase to the TiO_2_ anatase phase in the ErTiNTs [57]. As observed, in addition to tubular diameter and sodium content, oxygen vacancies have a strong influence on the optical properties of the samples studied. Thus, the Urbach energy was calculated and we were able to confirm the presence of these vacancies, which corroborates with the UV–Vis and PL absorption results.

## 4. Conclusions

This study described the synthesis, structure, morphology, and optical properties of ErTiNTs (NaTiNTs ion exchanged by Er^3+^) thermally treated in different atmospheres with the aim of engineering the electronic properties for optoelectronic applications. We observed changes in the characteristic Raman modes of the TiNTs, an increase in the interlamellar distance of the tubular structure of the ErTiNTs, the preservation of the 1D fibrillar morphology, and an accentuated decrease in the Na/Ti ratio with the increase in the Er/Ti ratio, suggesting the effectiveness of the ion exchange process. Furthermore, variations in the dimensions of TiNTs (diameter and interlamellar space) were observed with ion exchange and different thermal treatments. The insertion of Er^3+^ ions promoted a stabilization of the anatase phase during thermal treatment. In the optical properties evaluated by UV–Vis absorption spectroscopy and PL, the samples showed changes in optical absorption and photoluminescence emission, which were related to the changes in the electronic properties promoted by insertion of Er^3+^ ions and the different phases formed during the thermal treatment. Moreover, the Urbach energy revealed the formation of oxygen vacancies that directly influence the PL emission and the band gap energy of the samples. According to the results, the insertion of Er^3+^ ions associated with thermal treatment in an inert atmosphere can tune the optoelectronics and photonics properties of titanate nanotubes. Thus, calcined ErTiNTs in argon atmosphere showed superior improvement of their optical properties in relation to the other samples analyzed, being here presented as a promising material for applications in photoluminescent devices, displays, and lasers.

## Figures and Tables

**Table 1:** Samples of NaTiNT and ErTiNT heat treated in air (O_2_) and argon atmospheres presenting the atomic ratios Na/Ti and Er/Ti obtained by EDS.

Room Temperature			200 °C		400 °C		600 °C		800 °C	
Samples	Atomic Mean of the Samples (%)	Ratio Na or Er/Ti (%)	Na/Ti	Er/Ti	Na/Ti	Er/Ti	Na/Ti	Er/Ti	Na/Ti	Er/Ti
NaTiNT_O_2_	8.50	0.84	0.44	-	0.75	-	0.45	-	0.63	-
NaTiNT_Ar	8.50	0.84	1.03	-	0.74	-	0.64	-	0.11	-
ErTiNT_O_2_	6.20	0.30	0.27	0.12	0.0297	0.12	0.00	0.10	0.11	0.11
ErTiNT_Ar	6.20	0.30	0.14	0.11	0.09	0.10	0.00	0.11	0.16	0.11

## Data Availability

Not Applicable.

## Supplementary Materials

The following supporting information can be downloaded at: https://www.mdpi.com/article/10.3390/ma16051842/s1, Figure S1: The estimation of the band gap by Tauc plots of the NaTiNT_O_2_ and NaTiNT_Ar samples; Figure S2: The estimation of the band gap by Tauc plots of the ErTiNT_O_2_ and ErTiNT_Ar samples; Figure S3: Determination of Urbach energy of NaTiNT_O_2_ and NaTiNT_Ar samples; Figure S4: Determination of Urbach energy of ErTiNT_O_2_ and ErTiNT_Ar samples; Figure S5: Excitation PL spectrum (emission at 430 nm) of NaTiNTs and ErTiNTs calcined in air and argon atmosphere. The prefix “i” in figures (b) and (d) indicate the samples treated in inert atmosphere (argon). The insets were normalized and translated vertically. Variations in PL intensity occur with increasing temperature for all samples independently of the atmosphere; Figure S6: Changes (redshift and blueshift) in the positions of the peaks referent to the TiNT structure with the insertion of Er^3+^ ions and thermal treatment in air and argon atmosphere. The peaks referring to Er^3+^ ions are not presented.
