# Supplementary material for: Study of Structural and Optical Properties of Titanate Nanotubes with Erbium under Heat Treatment in Different Atmospheres
Source: Materials (Basel). 2023 Feb 23;16(5):1842. doi: 10.3390/ma16051842 (PMC10004321; doi:10.3390/ma16051842)
Supplement: Supplementary file 1 [file materials-16-01842-s001.zip › materials-2197073-supplementary.pdf]

Supplementary Material

Title: Synthesis of ErTiNTs, characterization and evaluation of structural and optical properties under heat treatment in different atmospheres

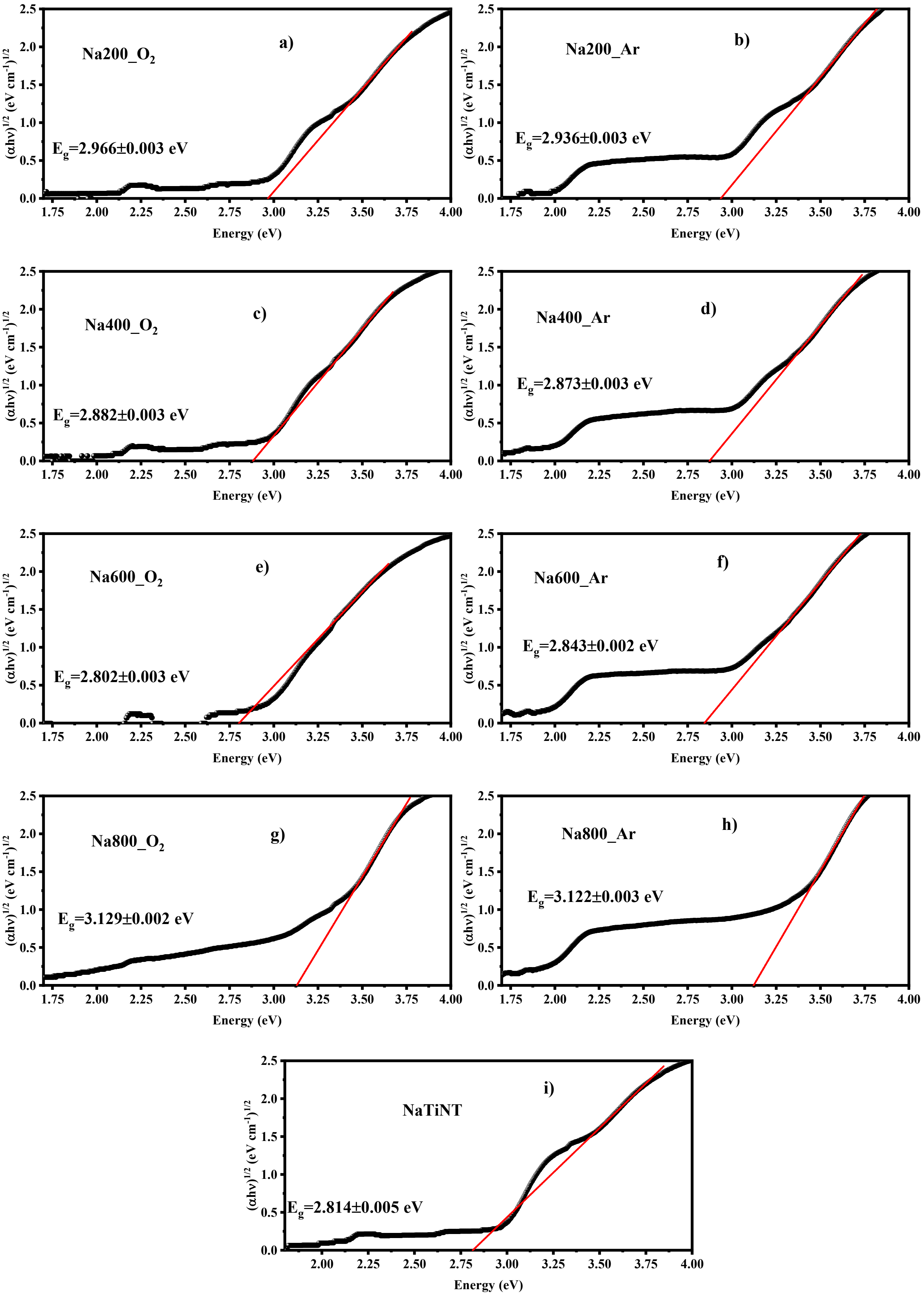

Figure S1: The estimation of the band gap by Tauc plots of the NaTiNT_O_2 and NaTiNT_Ar samples: a) Na200_Ar, b) Na200_O_2, c) Na400_O_2, d) Na400_Ar, e) Na600_O_2, f) Na600_Ar, g) Na800_O_2, h) Na800_Ar and i) NaTiNT.

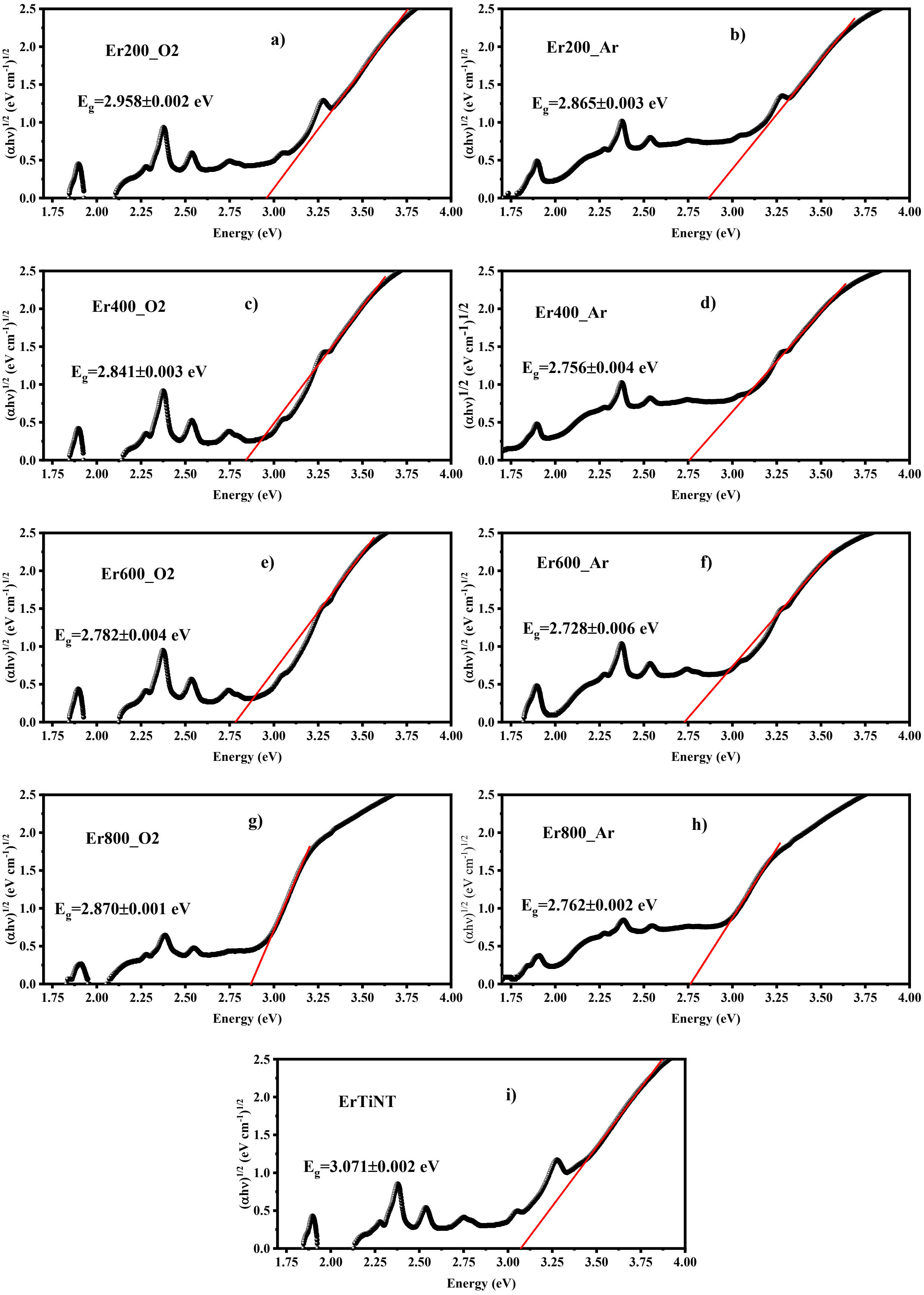

Figure S2: The estimation of the band gap by Tauc plots of the ErTiNT_O_2 and ErTiNT_Ar samples: a) Er200_Ar, b) Er200_O_2, c) Er400_O_2, d) Er400_Ar, e) Er600_O_2, f) Er600_Ar, g) Er800_O_2, h) Er800_Ar and i) ErTiNT.

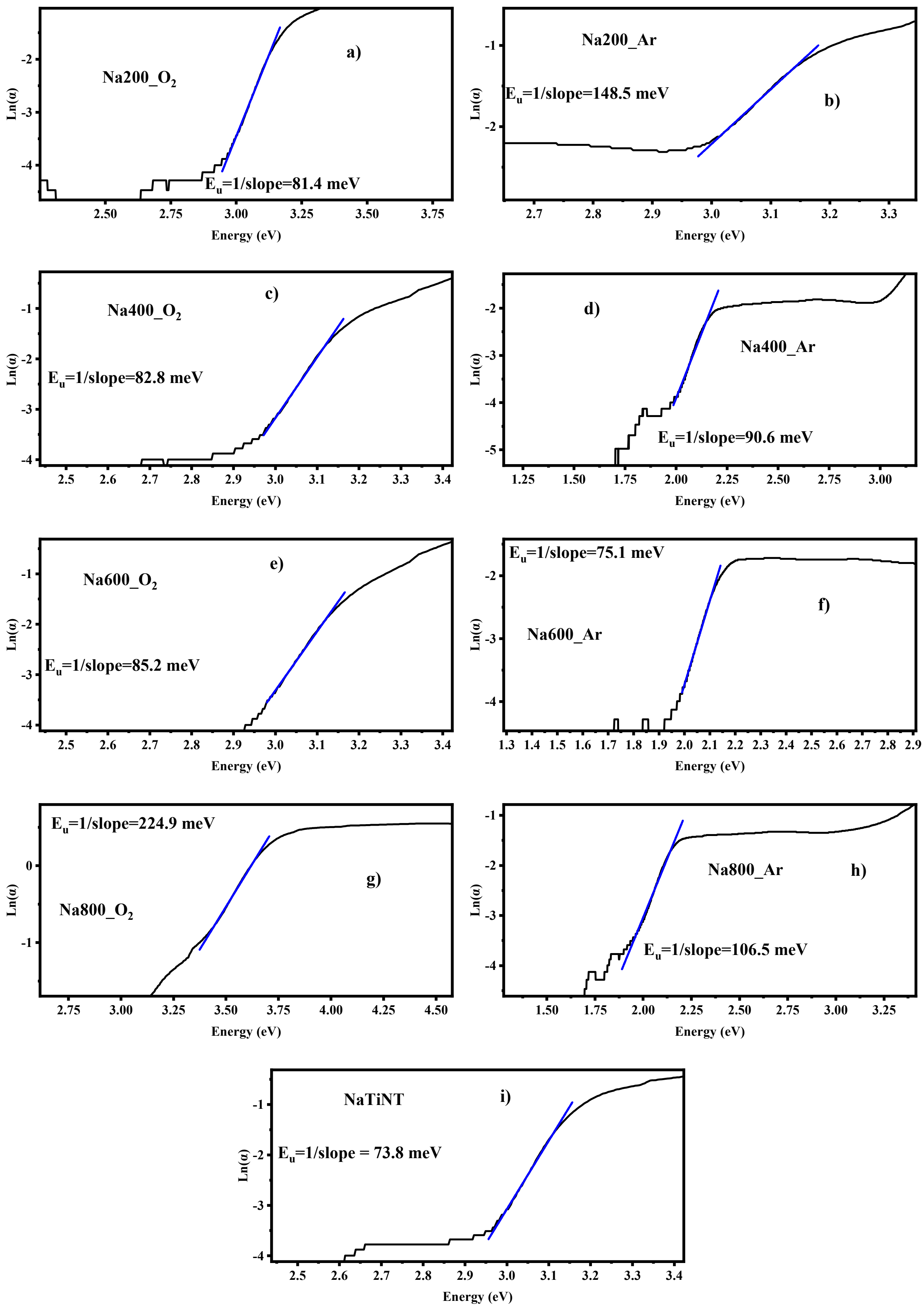

Figure S3: Determination of Urbach energy of NaTiNT_O2 and NaTiNT_Ar samples: a) Na200_Ar, b) Na200_O2, c) Na400_O2, d) Na400_Ar, e) Na600_O2, f) Na600_Ar, g) Na800_O2, h) Na800_Ar and i) NaTiNT.

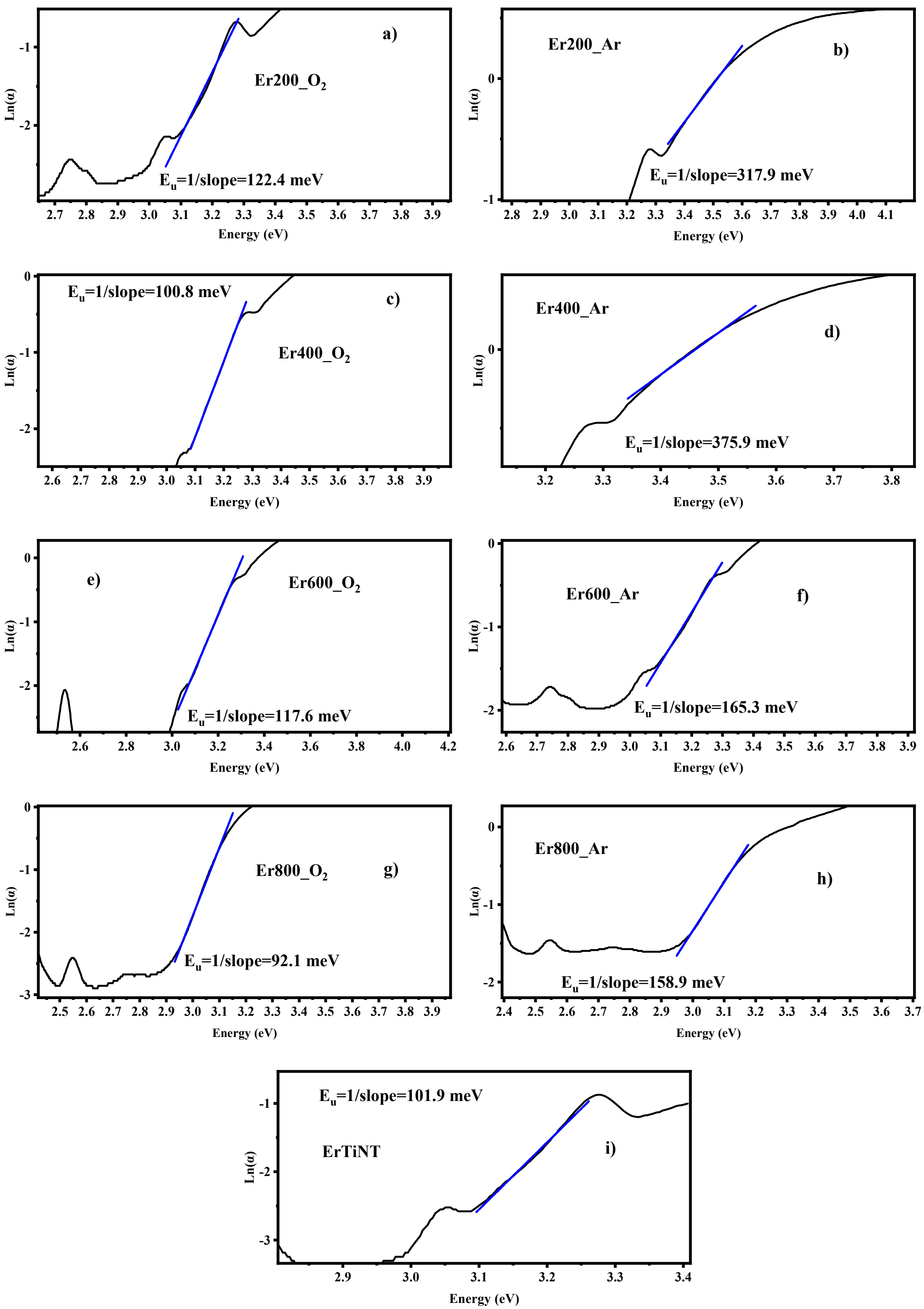

Figure S4: Determination of Urbach energy of ErTiNT_O2 and ErTiNT_Ar samples: a) Er200_Ar, b) Er200_O2, c) Er400_O2, d) Er400_Ar, e) Er600_O2, f) Er600_Ar, g) Er800_O2, h) Er800_Ar and i) ErTiNT.

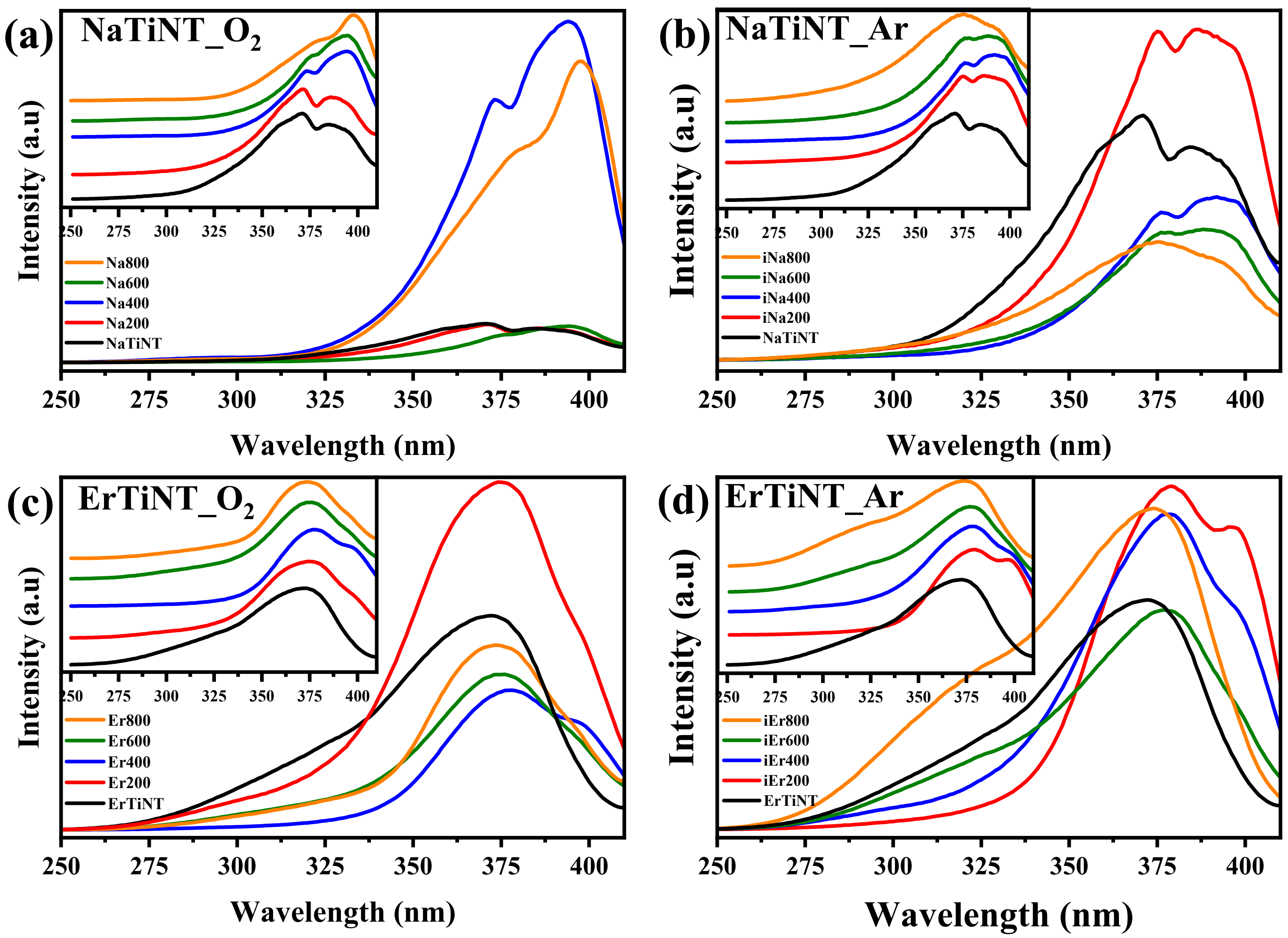

Figure S5: Excitation PL spectrum (emission at 430 nm) of NaTiNTs and ErTiNTs calcined in air and argon atmosphere. The prefix "i" in figures (b) and (d) indicate the samples treated in inert atmosphere (argon).The insets were normalized and translated vertically. Variations in PL intensity occur with increasing temperature for all samples independently of the atmosphere.

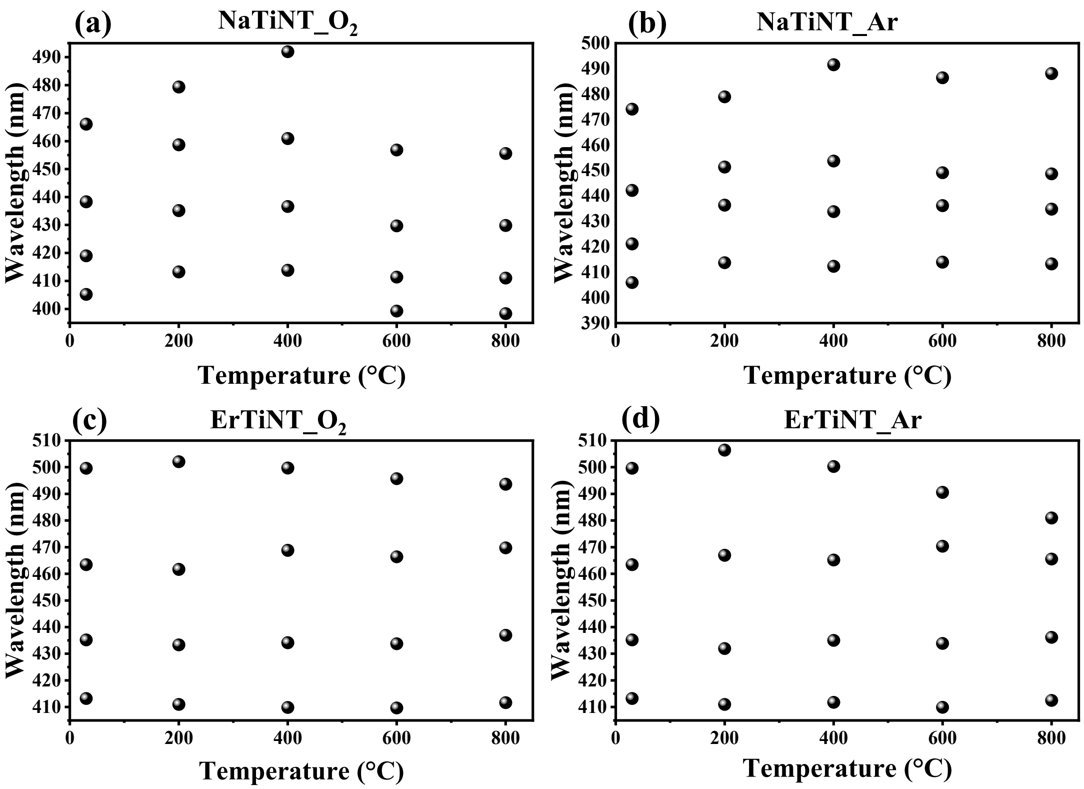

Figure S6: Changes (redshift and blueshift) in the positions of the peaks referents to the TiNT structure with the insertion of Er^{3+} ions and thermal treatment in air and argon atmosphere. The peaks referring to Er^{3+} ions are not presented.
